# Response of the authors regarding article “J point elevation in high precordial leads associated with risk of ventricular fibrillation”

**DOI:** 10.1111/anec.12871

**Published:** 2021-07-21

**Authors:** Yuki Hasegawa, Hiroshi Watanabe, Yasuhiro Ikami, Sou Otsuki, Kenichi Iijima, Nobue Yagihara, Daisuke Izumi, Tohru Minamino

**Affiliations:** ^1^ Department of Cardiovascular Biology and Medicine Niigata University Graduate School of Medical and Dental Sciences Niigata Japan; ^2^ Department of Cardiovascular Biology and Medicine Juntendo University Graduate School of Medicine Tokyo Japan

## CONFLICT OF INTEREST

None declared.

## AUTHOR CONTRIBUTIONS

All persons who meet authorship criteria are listed as authors, and all authors certify that they have participated sufficiently in the work to take public responsibility for the content, including participation in the concept, design, writing, or revision of the manuscript. Furthermore, each author certifies that this material or similar material has not been and will not be submitted to or published in any other publication before its appearance in the Annals of Noninvasive Electrocardiology.

## ETHICS STATEMENT

This study was approved by the ethics committee of Niigata University Medical & Dental Hospital.

We thank Dr. Karakus et al. for their interest in our recently published paper assessing the usefulness of electrocardiogram recordings in the high intercostal space to identify the risk of sudden death. (Hasegawa et al., 2020) In their letter to the editor, they expressed two concerns about the findings of our study. First, they suggested the need to include clinical data of modular factors including age, heart rate, electrolyte imbalance, hyperthermia, and usage of pharmacological agents on analysis for making clear the relationship between J point elevation in high precordial leads and ventricular fibrillation. These factors could influence the development of ventricular fibrillation in J wave syndrome, (Antzelevitch et al., 2017) and we agree with the importance of considering their influences. However, in our cohort, there was no patient showing abnormalities in electrolytes or body temperature. Regarding medications, as described in our paper, only two patients with Brugada syndrome received antiarrhythmic drugs, but in other ones did not have any drugs that can affect the J wave. We showed that there is no difference in the baseline heart rate in each group. Therefore, we subjected the other factors including J point elevation in the 3rd intercostal spaces, age, and sex at diagnosis to Cox proportional hazard analysis followed by stepwise forwards and backward regression modeling. We found that J point elevation in the 3rd intercostal spaces was significantly associated with the development of ventricular fibrillation in the patients with idiopathic ventricular fibrillation, as shown in the Table 1.

**TABLE 1:** Results of multivariate cox analysis

	Hazard ratio	95% CI	*p* value
Male sex	1.300	0.144–11.627	.815
Age	0.976	0.935–1.019	.270
J point elevation in the 3rd intercostal space	5.275	1.020–27.285	.047

Second, Karakus et al. pointed out the problems of the statistical measurement methods regarding the skewness in our cohort and the comparison between the three groups. We had confirmed that all of the parameters in three groups were normally distributed and used one‐way ANOVA for the comparisons.

## Data Availability

The data that support the findings of this study are available on request from the corresponding author. The data are not publicly available due to privacy or ethical restrictions.
